# All-Optical Assay to Study Biological Neural Networks

**DOI:** 10.3389/fnins.2018.00451

**Published:** 2018-07-05

**Authors:** Wardiya Afshar Saber, Federico M. Gasparoli, Marjet G. Dirks, Frank J. Gunn-Moore, Maciej Antkowiak

**Affiliations:** ^1^School of Medicine, University of St. Andrews, St. Andrews, United Kingdom; ^2^School of Biology, University of St. Andrews, St. Andrews, United Kingdom

**Keywords:** all-optical assay, optogenetics, calcium imaging, neural networks, connectivity, neurons, lentiviral transduction

## Abstract

We introduce a novel all-optical assay for functional studies of biological neural networks *in vitro*. We created a novel optogenetic construct named OptoCaMP which is a combination of a channelrhodopsin variant (CheRiff) and a red genetically encoded calcium indicator (GECI) (jRCaMP1b). It enables simultaneous optical stimulation and recording from large population of neurons with single-cell readout. Additionally, we have developed a spatio-temporal all-optical assay to simultaneously stimulate a sub-section of a neural network and record evoked calcium activity, in both stimulated and non-stimulated neurons, thus allowing the investigation of the spread of excitation through an interconnected network. Finally, we demonstrate the sensitivity of this assay to the change of neural network connectivity.

## Introduction

Functional studies of biological neural networks are fundamental to understand brain activity, to investigate how these networks are altered in brain disorders, and to help develop new treatments (Alivisatos et al., 2012). To explore heterogeneous neural networks, it is often critical to understand the activity of multiple neurons and how they communicate with each other. To capture the connectivity, it is essential to have a tool potentially applicable to both 2D and 3D neuronal culture systems that enables the simultaneous stimulation and recording from a large population of neurons with single-cell readout. Unfortunately, the traditional electrophysiology tools typically used are technically demanding and time consuming. Additionally, they exhibit severe limitations in terms of the number of studied cells, duration of the experiment and cellular viability (Scanziani and Hausser, 2009). Multi-electrode arrays offer the possibility to stimulate and record neuronal electrical activity from multiple neurons simultaneously (Gross et al., 1995); however, due to their design, they have a relatively low spatial resolution and do not guarantee single-cell resolution for either recording and/or stimulation.

Optogenetics is an emerging field that brings the possibility to overcome these limitations. It benefits from the synergy of optical and genetic techniques (Boyden et al., 2005) allowing contact-free manipulation of specific neurons and enabling repetitive interrogation of the same neurons. Optogenetic tools based on light-activated ion channels (Boyden et al., 2005) can be used to hyper or depolarise the cellular membrane using specific wavelengths of light (Tye and Deisseroth, 2012). A variety of these molecular tools, in particular channelrhodopsin variants, have been developed over the last decade in an effort to improve light sensitivity (Berndt et al., 2008, 2011), kinetics (Lin et al., 2009), and spectral response (Klapoetke et al., 2014).

More recently, Channelrhodopsins (ChRs) have been successfully combined with genetically encoded voltage indicators (GEVIs) (Lin and Schnitzer, 2016) to produce a purely optical readout of neuronal activity in a technique called optical electrophysiology (Hochbaum et al., 2014). This approach creates unprecedented possibilities for contact-free functional single-cell studies. However, due to the rapidity of the millisecond-scale neuronal electrical activity, high-fidelity recordings of large neuronal networks and the subsequent high-throughput functional characterization of the neurons require complex microscopy and high laser illumination intensities (Hochbaum et al., 2014).

While electrophysiological recordings of the membrane potential provide a direct and precise measure of neuronal activity, imaging the resulting changes in the intracellular calcium concentration is widely used as an indirect secondary reporter of activity as it is more easily captured using widely accessible fluorescence microscopy (McCombs and Palmer, 2008). This technique offers the possibility to simultaneously study large populations of neurons with single-neuron readout. Even though calcium imaging can be performed using calcium dyes (Bootman et al., 2013), this approach does not allow long-term studies, nor the targeting of specific cell types (Grienberger and Konnerth, 2012). In contrast, genetically encoded calcium indicators (GECIs) enable contact-free studies and allow to target different types of neurons in co-cultures by using specific promoters (Kim et al., 2017). Additionally, the use of specific promoters is an advantage for the study of networks with the ability to selectively stimulate a desired subset of neurons. In this paper, we use the capacity of channelrhodopsins to actively depolarize and evoke action potentials in cortical neurons (Nagel et al., 2003), and demonstrate that an approach based on the combination of optogenetics and calcium imaging, enables the evaluation of a network’s connectivity (Schoenenberger et al., 2011). Measuring the optically evoked calcium activity in neuronal cultures enables network activity studies, and could open new avenues in the understanding of network function (Packer et al., 2015). Previous attempts to combine ChRs with GECIs have shown limitations due to the excitation spectrum overlapping with those of light-activated ion channels such as channelrhodopsin-2 (ChR2) (Nagel et al., 2003). However, the development of GECIs with red-shifted excitation and emission spectra offers the possibility of combining it with a channelrhodopsin variant for spectrally independent photostimulation and imaging (Akerboom et al., 2012; Dana et al., 2016).

In this paper, we present OptoCaMP, a construct which enables contact-free, simultaneous stimulation and calcium recording of a large population of neurons *in vitro*. Additionally, we have designed an all-optical assay which enables the study of the spread of excitation, thus allowing quantification of network connectivity.

## Materials and Methods

### Design of OptoCaMP

OptoCaMP is a bicistronic vector for the co-expression of CheRiff-EGFP (Hochbaum et al., 2014) and the GECI jRCaMP1b (Dana et al., 2016). We used a 2A peptide ribosomal skip sequence as a means to achieve approximately stoichiometric co-expression of jRCaMP1b and CheRiff under the control of *CaMKIIα* promoter to target excitatory neurons (Morikawa et al., 2017)

The plasmid construct OptoCaMP (FCK-CaMKIIα-jRCaMP1b-P2A-CheRiff) was designed in our laboratory. NES-jRCaMP1b was amplified by PCR from the pGP-CMV-NES-jRCaMP1b plasmid (Addgene #63136) with the primers F-GAGAGA**GGATCC**ACCATGCTGCAGAACGAGCTT and R-GAGAGA**GGCGCGCC**CTACTTCGC TGTCATCATTTGTACAAACTC containing BamHI and AscI restriction sites, respectively. P2A-CheRiff was amplified by PCR from the FCK-Optopatch2 plasmid (Addgene #51694) with the primers F-GAGAGA**GGCGCGCC**GGCTCCGGAGCCACGAACTTC and R-GAGAGA**GAATTC**TTACTTGTACAGCTCGTCCATGCC containing AscI and EcoRI restriction sites, respectively. The amplified NES-jRCaMP1b and P2A-CheRiff were first digested with BamHI-AscI and AscI-EcoRI, respectively, and then cloned into a lentiviral vector FCK under the *CaMKIIα* promoter from the FCK-Optopatch2 plasmid (Addgene #51694) digested with BamHI and EcoRI.

FCK-Optopatch2 was a gift from Adam Cohen (Addgene plasmid # 51694) and pGP-CMV-NES-jRCaMP1b was a gift from Douglas Kim (Addgene plasmid # 63136).

### Neuronal Culture

Primary Rat Cortex Neurons obtained from Gibco^®^ by Life technologies^TM^ were isolated from day-18 Fisher 344 rat embryos and cryopreserved in a medium containing DMSO. Each vial contains 1 × 10^6^ viable neurons highly pure cells containing minimum number of astrocytes and other glial cells (>90% MAP2 positive live cells detected by immunofluorescence according to certificate analysis from Life technologies^TM^). The plating medium was Neurobasal^®^ supplemented with GlutaMAX^TM^-I to a final concentration of 0.5 mM and B27 Supplement. The primary rat cortex neurons were rapidly and gently thawed in a 37°C water bath and plated at a density of 2.10^5^/cm^2^ for a plated volume of 650 μl in WPI Fluorodish^TM^ glass bottom cell culture dishes coated with poly-D-lysine. The neurons were incubated at 37°C in a humidified atmosphere of 5% CO_2_. After 14 h of incubation, half of the medium from was replaced with fresh plating medium. Half of the media was replaced every third day with fresh complete Neurobasal^®^, B-27^TM^ Supplement and GlutaMAX^TM^-I to a final concentration of 0.5 mM or complete BrainPhys^TM^ and Neurocult^TM^ SM1 to a final concentration of 0.5 mM.

Neurons were transduced after 7 days *in vitro* (DIV) (Hochbaum et al., 2014) with OptoCaMP plasmid using a lentiviral transduction protocol (Ding and Kilpatrick, 2013). All experiments were performed at DIV 14–15 (Hochbaum et al., 2014). For synaptic transmission experiment, CNQX (2 μM) and D-APV (10 μM), respectively, AMPA and NMDA receptor antagonists (Lin et al., 2002) were added in Tyrode’s solution and incubated 30 min prior to the recordings. For caffeine experiments, caffeine (1 mM) (van Aerde et al., 2015) was added in Tyrode’s solution and incubated 30 min prior the recordings.

### Lentivirus Production and Transduction

Human Embryonic Kidney 293T/17 cell line obtained from ATCC^®^ is a derivative of the 293T cell line. The HEK 293T/17 were cultured in Dulbecco’s Modified Eagle’s Medium GlutaMAX^TM^-I supplemented with 10% Fetal Bovine Serum and 1% Penicillin/Streptomycin in 75 cm^2^ flasks at 37°C in a humidified atmosphere of 5% CO_2_. HEK 293T/17 were transfected using *Trans*IT^®^-LT1 Transfection Reagent with the lentiviral envelope vector *pSD11* (*VSV-G*) and packaging vector pSD16 to deliver 4 μg of FCK-OptoCaMP. Viral particles were collected, and neurons were transduced on DIV7 with OptoCaMP via lentiviral transduction. All OptoCaMP recordings were then performed in Tyrode’s salts (CaCl_2_2⋅H_2_O 0.26 g/L, MgCl_2_⋅6H_2_O 0.214 g/L, KCl 0.2 g/L, NaHCO_3_ 1 g/L, NaCl 8 g/L, NaH_2_PO_4_ (anhydrous) 0.05 g/L, D-Glucose 1 g/L) at DIV 14–17.

### Cytotoxicity Assay

Cytotoxicity in primary cortical neurons was measured at DIV 14 by lactate dehydrogenase (LDH) release assays (Pierce^TM^ LDH Cytotoxicity assay kit) as per the manufacturer’s protocol 7 days after OptoCaMP lentiviral transduction. Briefly, 50 μL of the supernatant is transferred to a 96-well plate and 50 μL of the reconstituted 2× LDH assay buffer were added to each sample medium and incubated for 30 min at room temperature protected from light. The maximum LDH activity was determined after treating neurons with lysis buffer as described in the manufacturer’s protocol. The absorbance was measured at 490 and 680 nm and the 680 nm absorbance value (background) was subtracted from the 490 nm absorbance.

### Microscopy Setup

The samples were imaged using an epifluorescence Nikon Ti inverted microscope, a Nikon 20× NA 0.75 air objective and a sCMOS Camera (Zyla 5.5, Andor). This setup allows for a field of view of 640 × 540 pixels with 4 × 4 pixel binning. The blue illumination for the stimulation of the light-activated ion channel CheRiff, was provided by a blue light LED at 470 nm (Thorlabs M470L3 mounted LED) through a B2A filter set (no emission filter, excitation filter 450–490 nm). A lever-actuated iris diaphragm (Thorlabs SM1D12) with a XY translating lens mount (Thorlabs LM1XY/M) was installed after the blue LED in a plane conjugated with the focal plane of the microscope objective. In this way, within the field of view, different sizes and/or positions of the blue illumination could be selected. The change in fluorescence from the GECI jRCaMP1b was recorded at 10 Hz under continuous green illumination at 550 nm through a filter set (excitation filter 565/24 nm, dichroic mirror 562 nm, longpass emission filter >570 nm). A National Instruments LabVIEW 14.0 (Elliott et al., 2007) program was developed in our laboratory and used to create a voltage output that results in a modulation of the blue light intensity under continuous green illumination. The relationship between the voltage output and the blue and green illuminations was calibrated using a power meter with Photodiode Sensor (Thorlabs). The intensity at the sample was calculated following the calibration and according to the Nikon 20× NA 0.75 air objective used.

### Optical Stimulation and Calcium Recordings

In a typical experimental procedure, the field of view was chosen using the 20× objective. The protocol applied consisted in steps of increasing blue light intensity (corresponding to 10% of the LED power) equal to 7 mW/cm^2^ (± 1 mW/cm^2^) measured at the sample from 0 to 67 mW/cm^2^. Each recording consisted of 10 pulses of blue light (470 nm) applied to a defined area or to the whole field of view. Pulses duration chosen were 10, 100, 250, and 500 ms and each pulse was separated by an interval of 5.5 s. Recordings were performed under continuous green illumination (550 nm, 7% corresponding to 15 mW/cm^2^ measured at the sample) at 10 frames/s for a total duration of 1 min per field of view (600 frames). The protocols were externally triggered through the LabVIEW script designed in our laboratory and the image acquisition was simultaneously performed through the Andor Solis software (Andor Technology, Belfast, United Kingdom) (Usami et al., 2008).

### Data Processing and Statistical Analysis

Following the acquisition, the recordings were processed using a Matlab^®^ script designed in our laboratory and a Matlab^®^ toolbox called NeuroCa (Jang and Nam, 2015). This process consists of cell body detection using Circular Hough Transform and the Matlab^®^ function *imfindcircles*. After segmentation, each soma is considered as a region of interest (ROI). To correct for photobleaching and calculate *ΔF/F*, we used NeuroCa to estimate the baseline *F* for each calcium signal by double curve fitting with the exponential decay function (Jang and Nam, 2015). Following this step, we obtain the calcium signals with zero baseline for each ROI (each neuron). For analysis purposes and to select the more robust protocol for further assays, we divided the field of view recorded into three zones. In the green and red zones (zones 1 and 2, respectively) neurons were not stimulated with blue pulses of light. The discrimination between these two zones was defined by drawing a virtual circle of 600 μm which corresponds to two times the radius of the blue zone (300 μm). We extracted the peak fluorescence of individual neurons and calculated the mean ± SEM of the neurons for each zone. The mean global connectivity and the connectivity maps are obtained using the software FluoroSNNAP, an open-source program for the automated quantification of calcium dynamics of single-neurons and network connectivity (Patel et al., 2015). After segmentation, each soma is considered as a ROI (Supplementary Figure 3). Initially, several measurements such as the amplitude, the rise time and fall time of the calcium events are used to determine the spatiotemporal profile of calcium activity within individual neurons (Supplementary Figure 3). The amplitude is defined as the maximum peak fluorescence intensity over baseline following the onset of a calcium event. The rise time is determined as the time-to-peak fluorescence intensity and the fall time is defined with the exponential decay of the curve fitting the calcium event. These measurements are used to determine temporal relationships between the time series of two neurons, *x*(*t*) and *y*(*t*), through cross-correlation. The network connectivity is then quantified using the mean global connectivity, based on a cross-correlation between pair-wise fluorescence traces reshuffled 100 times for statistical significance. The mean global connectivity is defined as:

$$\begin{array}{c} \frac{mean(sum(A))}{N-1} \end{array}$$

where *A* is a binary connectivity matrix, *A*(*i,j*) = 1 indicates connection between *i* and *j* nodes and *A*(*i,j*) = 0 indicates absence of connection. The average of the number of connections across all nodes divided by the total number of nodes is the mean global connectivity. A graph where every node is connected to every other node will have mean global connectivity of 1. Data were expressed as means ± SEM. and one-way or two-way ANOVAs tests were used as indicated in the figure legends.

## Results

### OptoCaMP: A Novel Optogenetics Construct for All-Optical Neuronal Activity Studies

**FIGURE 1:**
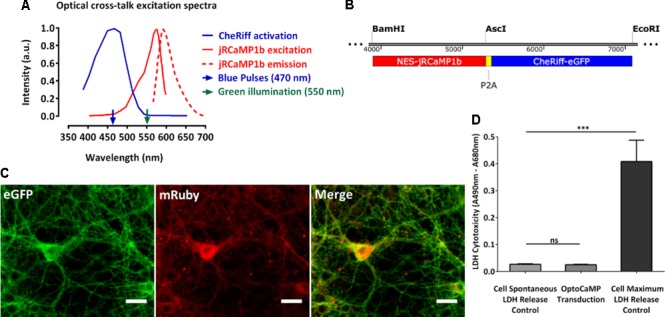
OptoCaMP enables simultaneous stimulation and calcium recording in neuronal dissociated cultures. **(A)** Activation spectrum of CheRiff (blue) and excitation/emission spectra of jRCaMP1b (red). The green and blue arrows, respectively, indicate the continuous green illumination at 550 nm and the blue pulses at 470 nm. **(B)** Schematic of OptoCaMP, bicistronic lentiviral construct designed for the stoichiometric co-expression of the Genetically Encoded Calcium Indicator (GECI) jRCaMP1b (red box) and the Channelrhodopsin variant CheRiff (blue box) to enable simultaneous stimulation and calcium imaging. **(C)** Single channels and merged fluorescence images of rat cortical neurons expressing OptoCaMP at 14 days *in vitro:* CheRiff-eGFP is displayed in green and mRuby based GECI jRCaMP1b in red. Scale bar 25 μm. **(D)** Quantification of lactose dehydrogenase levels (LDH) in primary cortical neurons 6 days after transduction with the OptoCaMP. The lentivirus delivery did not show any significant difference between the cells non-transduced (cell spontaneous LDH release control) and the cell transduced with OptoCaMP. (*w =* 3, one-way ANOVA, *F*(2,6) = 69.49, *P* < 0.0001; followed by Tukey’s multiple comparisons test: OptoCaMP transduction versus Cell spontaneous LDH release control, *P =* 0.992; Cell maximum LDH release control versus Cell spontaneous LDH release control, ^∗∗∗^*P* < 0.0001; Cell maximum LDH release control versus OptoCaMP transduced, ^∗∗∗^*P* < 0.0001).

We created OptoCaMP as a combination of the blue light-activated ion channel CheRiff (Hochbaum et al., 2014) and the GECI jRCaMP1b (Dana et al., 2016). CheRiff, a channelrhodopsin variant, is activated under blue illumination at 470 nm and demonstrates rapid kinetics (Hochbaum et al., 2014). We combined it with the mRuby-based GECI jRCaMP1b which has a larger dynamic range compared to other red-GECIs. Additionally, jRCaMP1b does not show saturation in the range of 1–160 action potentials nor photo-switching after illumination with blue light (470 nm, 3200 mW/cm^2^) (Dana et al., 2016). The mRuby based GECI jRCaMP1b spectra do not exhibit overlap with the peak excitation of CheRiff (λ_excitation_ = 460 nm) thus avoiding optical cross-talk (**Figure 1A**). Additionally, we supported experimentally that the continuous green illumination did not affect the resting membrane potential of the neurons expression OptoCaMP (Supplementary Figure 2) confirming that our combination enables simultaneous optical stimulation and calcium imaging (Ca^2+^ imaging). We used a 2A peptide ribosomal skip sequence to achieve stoichiometric co-expression of jRCaMP1b and CheRiff (**Figure 1B**). The nuclear export sequence (NES) was present to the N-termini of jRCaMP1b to restrict its expression in the cytoplasm. We used a *CaMKIIα* promoter to express OptoCaMP in excitatory primary rat cortex neurons (Yaguchi et al., 2013; Morikawa et al., 2017) through a lentiviral delivery protocol (Ding and Kilpatrick, 2013; Packer et al., 2013) (**Figure 1C**) and additionally confirmed the integration of the two fragments CheRiff and jRCaMP1b with a restriction digestion of the OptoCaMP (Supplementary Figure 1). We tested the cytotoxicity of the lentivirus OptoCaMP by quantifying the LDH release as an indicator of cell membrane integrity (Björk et al., 2017) 7 days after transduction (DIV 14). This assay did not show any significant difference between the LDH release of the non-transduced neurons (cell spontaneous LDH release control) and the transduced neurons (OptoCaMP transduction.) (**Figure 1D**).

### OptoCaMP Enables Simultaneous Stimulation of a Large Neuronal Population With Single-Neuron Readout

**FIGURE 2:**
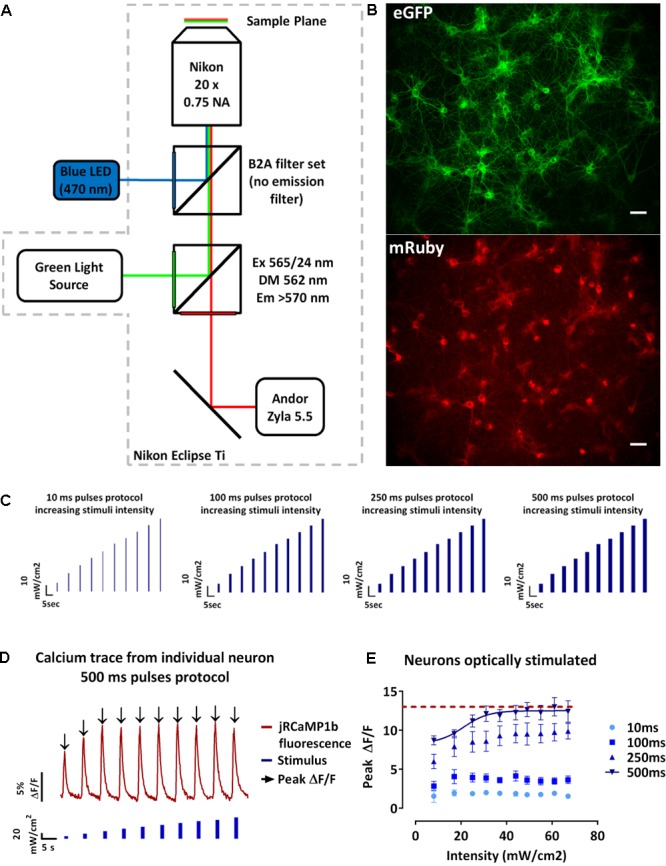
Optical recordings of light evoked-calcium event in neuronal dissociated cultures. **(A)** Schematic drawing of the imaging setup for all-optical stimulus and readout. The setup is based on a Nikon Eclipse Ti epi-fluorescence microscope mounting a B2A filter set (without the emission filter) for the blue LED illumination (470 nm) and a modified Cy3/TRITC longpass filter set (excitation filter 565/24 nm, dichroic mirror 562 nm, longpass emission filter >570 nm) for the green light source. Recordings are performed at 10 Hz using a Andor Zyla 5.5 sCMOS Camera. **(B)** Neuronal dissociated cultures at 14 days *in vitro* expressing OptoCaMP via lentiviral transduction. CheRiff-eGFP expression in green (eGFP) and mRuby based GECI jRCaMP1b in red (mRuby). Scale bar 100 μm. **(C)** Schematic of the temporal optical stimuli protocols which consisted in 10 consecutive pulses of blue light (470 nm) of 10 ms, 100 ms, 250 ms, and 500 ms with increasing steps of 7 mW/cm^2^± 1 mW/ cm^2^ between each pulse (measured at the sample). **(D)** Typical trace of the fluorescence change of the GECI jRCaMP1b (red trace) of a single-neuron during the 500 ms pulses protocol of increasing blue stimulation intensities (0 to 67mW/cm^2^). From this trace we can then extract the peak fluorescence ΔF/F (black arrow) for each stimulus intensity for individual neurons of the field of view simultaneously. **(E)** Amplitude of the calcium events in response to increasing stimuli intensities and pulse duration of 10, 100, 250, and 500 ms. The peak fluorescence from individual neuron at each stimuli intensity was averaged to obtain mean ± s.e.m. values (*n =* 3, two-way ANOVA, effect of the stimulus intensity *F*(9,560) = 41.80, *P* < 0.0001, effect of the stimulation duration *F*(3,560) = 4470, *P*<0.0001 followed by Tukey’s multiple comparisons test showing a plateau (red dashed line) for 500 ms pulses protocol, stimulus above 25 mW/cm^2^).

As previously described, the Channelrhodopsin variant CheRiff and the GECI jRCaMP1b have been reported to reliably evoke single action potentials following optical stimulation with blue light (470 nm, 10 ms, 8 mW/cm^2^) (Hochbaum et al., 2014) and to report single action potential up to 160 times without saturation for the GECI (Dana et al., 2016; Vogt, 2016). We initially wanted to test whether OptoCaMP would maintain these abilities. Therefore, we designed an experimental setup (Materials and Methods, **Figure 2A**) to stimulate CheRiff which elicits membrane depolarization and record the subsequent Ca^2+^ flux in a large neuronal population. The field of view recorded (832 μm × 702 μm), typically containing approximately 60 neurons expressing OptoCaMP (**Figure 2B**). We designed protocols based on a range of blue pulse duration from 10 ms to evoke a single action potential (Hochbaum et al., 2014), to 100, 250, or 500 ms duration to evoke a train of action potentials. CheRiff has previously been demonstrated to have a greatly increased sensitivity at low illumination intensity when compared to previously published channelrhodopsin variants (Hochbaum et al., 2014). We designed our protocols where ten steps of increasing light intensity of 7 mW/cm^2^ (± 1 mW/cm^2^) were applied from 0 to 67 mW/cm^2^ to validate the sensitivity of OptoCaMP and find the optimum illumination conditions (**Figure 2C**). These protocols were inspired by the traditional depolarizing current-injection protocols used in electrophysiology, where increasing current steps are applied to depolarize the neuron and determine a threshold for action potential initiation (Perkins, 2006). Each pulse was separated by 5.5 s intervals where no stimulation was applied to allow calcium concentration to decay to baseline levels. The signal processing of the change of fluorescence of jRCaMP1b (section “Materials and Methods” – Data processing and statistical analysis) resulted in single-neuron calcium traces (**Figure 2D**). To compare the activity during the protocols performed (**Figure 2C**), we extracted the peak fluorescence of single neurons and calculated the mean ± SEM of the neurons in the field of view. This analysis shows that the peak fluorescence intensities significantly increased with the pulse duration exhibiting the lowest calcium influx activity for the 10 ms pulses stimulation and the highest calcium influx with the 500 ms pulses protocol (*p* ≤ 0.0001) (**Figure 2E**). Additionally, we observed a plateau for the 500 ms pulses protocol by fitting a sigmoidal function to the data points suggesting that the maximum amplitude of calcium is reached for stimuli intensities above 25 mW/cm^2^ (**Figure 2E**). Moreover, the two-way ANOVA test followed by Tukey’s multiple comparisons test revealed that the peak fluorescence intensities recorded during stimuli intensities above 25 mW/cm^2^ were not significantly different from each other. Our results confirmed that OptoCaMP enables simultaneous optical stimulation and monitoring of neuronal activity.

### OptoCaMP Enables the Study of the Spread of Excitation in a Neural Network

**FIGURE 3:**
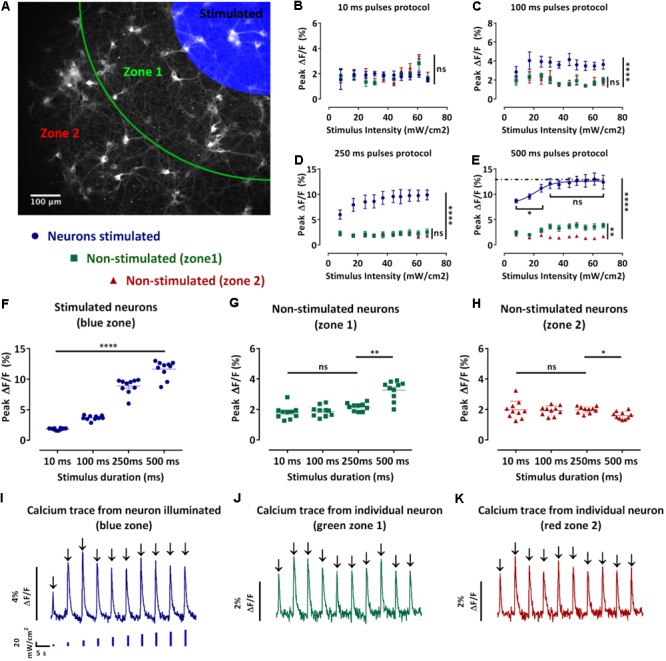
Design and analysis of the spatio-temporal optical stimuli protocols. **(A)** Map defining the three zones for the spatio-temporal optical stimuli protocols and analysis. Neurons in the blue zone were stimulated, neurons in zone 1 (green) and 2 (red) were not stimulated. **(B–E)** Average of the peak fluorescence intensities of single-neurons in the same zone ± s.e.m. against the stimulus intensity for the 10 ms **(B)**, 100 ms **(C)**, 250 ms **(D)**, and 500 ms **(E)** pulses protocols. According to the map in **(A)**, in blue, average peak fluorescence intensity for the stimulated neurons, in green, non-stimulated neurons in the zone 1 and in red, non-stimulated neurons in the zone 2 (*n =* 3 independent cell culture experiments, one-way ANOVA, **(B)** *F*(2,27) = 0.4533, *P* = 0.6403; **(C)** *F*(2,27) = 298.8, *P* < 0.0001; **(D)** *F*(2,27) = 301.7, *P* < 0.0001; **(E)** two-way ANOVA, effect of the stimulus intensity *F*(9,370) = 39.65, *P* < 0.0001 and effect of distance to stimuli (zones) *F*(2,370) = 9316, *P* < 0.0001; **(B–E)** followed by Tukey’s multiple comparisons test: ns=non-significant, ^∗^*P* < 0.05, ^∗∗^*P* < 0.01, ^∗∗∗∗^*P* < 0.0001). **(F–H)** Pooled peak fluorescence intensities of each defined zone separately ± s.e.m. **(F)** stimulated neurons, **(G)** non-stimulated neurons in zone 1, and **(H)** non-stimulated neurons in zone 2 for stimuli intensities from 0 to 67 mW/cm^2^ against the pulse duration. (*n=*3 independent cell culture experiments, two-way ANOVA, **(F)** effect of the stimulus intensity *F*(9,520) = 2810, *P* < 0.01 and effect of the stimulation duration *F*(3,520) = 296.1, *P* < 0.0001; **(G)** effect of the stimulus intensity *F*(9,520)=0.7990, non-significant and effect of the stimulus duration *F*(3,520) = 26.21, *P* < 0.0001; **(H)** effect of the stimulus intensity *F*(9,520) = 0.8480, non-significant and effect of the stimulus duration *F*(3,520) = 2.994, *P* < 0.05; **(F–H)** followed by Tukey’s multiple comparisons test: ns=non-significant, ^∗^*P*<0.05, ^∗∗^*P* < 0.01, ^∗∗∗^*P*<0.001, ^∗∗∗∗^*P*<0.0001). **(I–K)** Typical traces of the fluorescence change of the GECI jRCaMP1b of a single-neuron during the 100 ms pulses protocol of increasing blue stimulation intensities (0 to 67mW/cm^2^). **(I)** neuron stimulated; **(J)** non-stimulated neuron in zone 1 and **(K)** non-stimulated neuron in zone 2. From these traces we can then extract the peak fluorescence ΔF/F (black arrow).

We next developed an assay to achieve spatially selective optical excitation of a sub-section of the network, while the activity of the neighboring neurons along with the stimulated neurons was monitored. This assay allowed us to investigate the spread of excitation in the studied network. To achieve spatial stimuli, the previously described blue light pulses protocols (**Figure 2C**) were applied to a defined region in the field of view. For analysis purposes, as shown in **Figure 3A**, the field of view was divided into three zones: the blue zone containing the stimulated neurons and the zones 1 and 2, respectively, green and red, containing the non-stimulated neurons (section “Materials and Methods” – Data processing and statistical analysis). We then collected traces of the changes of jRCaMP1b fluorescence of individual neurons (**Figures 3I–K**), extracted the values of the peak fluorescence intensities following each stimulus and plotted the average ± SEM for the neurons localized in the same zones. The purpose of this study was to select a candidate protocol for further assays.

During the 10 ms pulses protocol, we observed an increase in the peak fluorescence for stimuli intensities between 44 and 60 mW/cm^2^ for the non-stimulated neurons in the zones 1 and 2 (**Figure 3B**). Moreover, this increase resulted in higher peak fluorescence intensities in the non-stimulated neurons compared to the stimulated neurons. However, there was no significant difference between the peak fluorescence in the non-stimulated neurons in zones 1 and 2 (**Figure 3B**). During the 100 ms pulses protocol, we observed that the peak fluorescence intensities for the stimulated neurons were significantly higher than the non-stimulated neurons in the zones 1 and 2 (*p* ≤ 0.0001) (**Figure 3C**). The peak fluorescence intensities were not significantly different for the non-stimulated neurons in the zones 1 and 2 (**Figure 3C**). During the 250 ms, the peak fluorescence intensities for the stimulated neurons increased with the stimuli intensities and were significantly higher than the peak fluorescence intensities for the non-stimulated neurons zones 1 and 2 (*p* ≤ 0.0001) (**Figure 3D**). In contrast, the peak fluorescence intensities of zones 1 and 2 were not significantly different (**Figure 3D**). During the 500 ms protocol, the peak fluorescence intensities for the stimulated neurons increased with the stimuli intensities until reaching a plateau for stimuli intensities above 25 mW/cm^2^ (**Figure 3E**). Furthermore, the peak fluorescence intensities of the stimulated neurons were significantly higher than the peak fluorescence for the non-stimulated neurons in zones 1 and 2 (**Figure 3E**). In contrast to the shorter pulses protocol where we did not observe any significant difference for the non-stimulated neurons between zones 1 and 2, during the 500 ms protocol we observed significantly lower peak fluorescence intensities for the neurons in zone 2 compared to the zone 1 (*p* ≤ 0.0001) (**Figure 3E**). We also observed that an increase in the pulse duration resulted in a significant increase in the peak fluorescence intensities for the stimulated neurons (*p* ≤ 0.0001) (**Figure 3F**). Regarding the non-stimulated neurons (zones 1 and 2), an increase in the pulse duration did not result in a significant difference during the 10, 100, and 250 ms protocol (**Figures 3G,H**). In contrast, we observed a significant increase in the peak fluorescence intensities for the non-stimulated neurons in the zone 1 during the 500 ms pulses protocol compared to the other protocols (*p* ≤ 0.01) (**Figure 3G**). We also observed a significant decrease in the peak fluorescence intensities for the non-stimulated neurons in the zone 2 during the 500 ms pulses protocol compared to the other protocols (*p* ≤ 0.05) (**Figure 3H**).

In summary, the two-way ANOVA test revealed a significant effect of the pulse duration and intensity on the calcium activity of the stimulated neurons. However, we only observed a significant effect of the stimulus duration but not stimulus intensity on the non-stimulated neurons zones 1 and 2. Although an increase in the pulse duration evoked greater Ca^2+^ response in the stimulated neurons, it did not result in greater Ca^2+^ activity in the non-stimulated neurons (**Figures 3F–H**). This phenomenon is potentially linked to the maximum synaptic activation between stimulated and non-stimulated neurons being reached. We also observed a significant reduction of the non-stimulated neurons Ca^2+^ response in the zone 2 for the longest pulse duration (**Figure 3H**). Moreover, high levels of intracellular calcium have been associated to cell damage (Mark et al., 2001), leading us to conclude that the 500 ms pulses was not the optimum stimulation. In contrast, the 10, 100, and 250 ms pulses protocols evoked significantly higher peak fluorescence intensities in the stimulated neurons without evoking significantly higher peak fluorescence intensities for the non-stimulated neurons in the zones 1 and 2. Both these protocols (100 and 250 ms pulses) demonstrated a better reproducibility than 10 ms so we selected the 100 ms pulses as the final protocol to achieve a higher throughput of our system.

### OptoCaMP Enables the Study of Neural Network Connectivity

**FIGURE 4:**
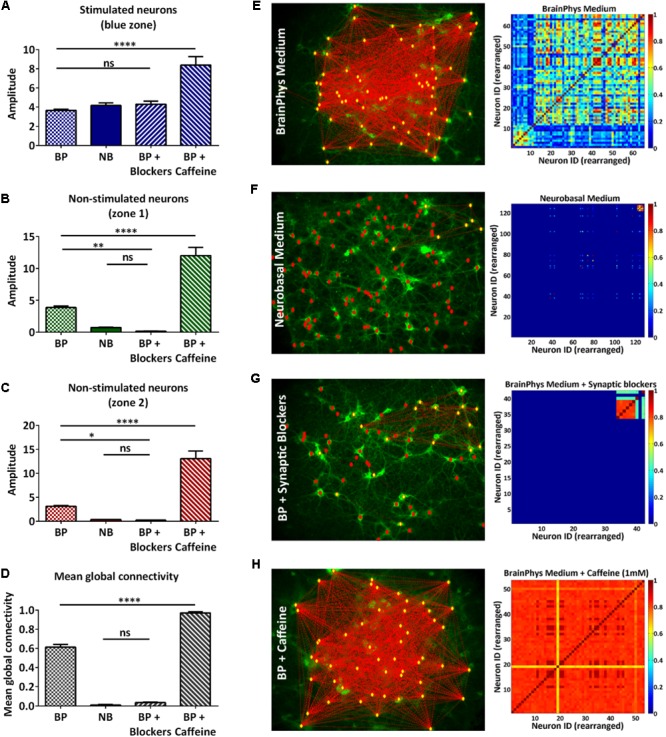
Qualitative and quantitative analysis of neural network connectivity. **(A–C)** Average of the amplitude of the calcium events of multiple individual neurons in the same zone ± s.e.m.: stimulated neurons **(A)**, non-stimulated neurons in zone 1 **(B)**, and zone 2 **(C)** during the 100 ms pulses protocol in four conditions: BrainPhys complete medium (BP), complete Neurobasal medium (NB), synaptic blockers (BP + blockers) and caffeine (BP + caffeine) (*n=*3 independent cell culture experiments for each condition; one-way ANOVA, **(A)** *F*(3,36) = 20.16, *P* < 0.0001; **(B)** *F*(3,36) = 66.31, *P* < 0.0001 **(C)** *F(*3,36) = 59.77, *P* < 0.0001; **(A–C)** followed by Tukey’s multiple comparisons test: ns=non-significant, ^∗^*P*<0.05, ^∗∗^*P*<0.01, ^∗∗∗∗^*P*<0.0001). **(D)** Mean global connectivity in BrainPhys complete medium (BP), complete Neurobasal medium (NB), synaptic blockers (BP + blockers) and caffeine (BP + caffeine) conditions. (*n* = 3 independent cell culture experiments for each condition; one-way ANOVA, *F*(3,8) = 854, *P*<0.0001; followed by Tukey’s multiple comparisons test: ns=non-significant, ^∗∗∗∗^*P* < 0.0001 **(E–H)** Analysis – Connectivity maps and colormaps. Connectivity of the whole network in complete BrainPhys medium **(E)**, complete Neurobasal medium **(F)**, complete BrainPhys medium with caffeine **(G)** and complete BrainPhys medium with blockers **(H)** conditions.

We have demonstrated that the all-optical functional assay we have developed, based on temporal and spatial stimulation, enabled the study of a neural network with a single-neuron readout. Quantifying the connectivity of the network would allow studies in disrupted or enhanced network connectivity conditions. To verify that our assay is sensitive to such changes, we designed a proof-of-concept experiment where we performed our all-optical functional assay using the 100 ms pulses protocol in dissociated neuronal cultures under four medium conditions (**Table 1**). The first condition represented the control experiment where the rat cortical neurons were maintained in complete BrainPhys medium which has been recently developed to support *in vitro* neuronal activity (Bardy et al., 2015). In the second condition, the rat cortical neurons were maintained in complete Neurobasal medium, a commonly used medium for neuronal cultures *in vitro* which has recently been linked to a reduction of synaptic communication and action potential firing compared to BrainPhys medium (Bardy et al., 2015). In the third condition, the neurons were maintained in BrainPhys basal medium and the synaptic blockers CNQX (6-Cyano-7-nitroquinoxaline-2,3-dione) and D-APV [D(-)-2-Amino-5-phosphonopentanoic acid], respectively, AMPA and NMDA receptors antagonists were added to the recording solution (Lin et al., 2002). In both Neurobasal and BrainPhys plus synaptic blocker conditions, we expected to see a reduced synaptic activity when compared to the BrainPhys condition. In the fourth condition, the neurons were maintained in BrainPhys basal medium and caffeine (1 mM) was added to the recording solution. Caffeine is an antagonist of the adenosine A1 and A2A receptors which has been shown to increase the excitability of neurons (Yanovsky et al., 2006; van Aerde et al., 2015) and excitatory synaptic transmission (Kerkhofs et al., 2018). For the latter condition, we expected an enhancement in neuronal activity in both the stimulated and non-stimulated neurons resulting in a higher mean global connectivity compared to the BrainPhys medium condition. As previously described (section “Materials and Methods,” **Figures 2C, 3A**, and Supplementary Figure 3), to compare the neuronal activity in each zone, we performed the analysis and collected the amplitude of each event in each neuron during the increasing blue light intensity 100 ms pulses protocol, and then we averaged the response of all the neurons within the same zone (**Figures 4A–C**). Regarding the stimulated neurons, we did not observe any significant difference between the BrainPhys medium, Neurobasal medium and BrainPhys medium with the addition of synaptic blockers (**Figure 4A**). Regarding the non-stimulated neurons, in zone 1 (green – **Figure 4B**) and zone 2 (red – **Figure 4C**), we did not observe a significant difference between the Neurobasal medium and BrainPhys medium with synaptic blockers; however, the neuronal response was significantly decreased compared to the BrainPhys medium condition (*p* ≤ 0.01 for zone 1 and *p* ≤ 0.05 for zone 2) (**Figures 4B,C**), which agrees with previous studies indicating that synaptic transmission is affected in Neurobasal (Bardy et al., 2015) and in BrainPhys medium with synaptic blockers (Lin et al., 2002). In contrast, the addition of caffeine resulted in a significant higher amplitude compared to the three other conditions in both stimulated (**Figure 4A**) and non-stimulated neurons (**Figures 4B,C**). These observations correlate with previous studies which have shown that caffeine enhances the excitability of neurons and excitatory synaptic transmission (*p* ≤ 0.0001) (van Aerde et al., 2015; Kerkhofs et al., 2018).

**Table 1:** Summary of the conditions studied.

Condition	Culture medium	Substance added to the recording solution	Expected effect of the condition
BrainPhys	Complete BrainPhys	Tyrode’s solution	Supports synaptic function (Bardy et al., 2015)
Neurobasal	Complete Neurobasal		Reduced synaptic communication (Bardy et al., 2015)
BrainPhys + synaptic blockers	Complete BrainPhys	Tyrode’s solution + CNQX and D-APV	Block synaptic communication (Lin et al., 2002)
BrainPhys + caffeine		Tyrode’s solution + Caffeine	Increases the excitability of neurons (Yanovsky et al., 2006; van Aerde et al., 2015)

*For the BrainPhys and Neurobasal conditions, the dissociated rat cortical neurons were, respectively, cultured in these complete media (see section “Materials and Methods”). Experiments for these two conditions were performed in the same Tyrode’s recording solution. For the BrainPhys with synaptic blockers and Brainphys with caffeine, neurons were cultures in complete BrainPhys medium. Experiments were performed in Tyrode’s solution, respectively, with the addition of CNQX (6-Cyano-7-nitroquinoxaline-2,3-dione) (2 μM) and D-APV (D(-)-2-Amino-5-phosphonopentanoic acid) (10 μM) or caffeine (1 mM).*

In addition, the calcium activity of individual neurons can be temporally correlated to characterize the network connectivity (Junek et al., 2009). As described in the section “Materials and Methods” (Data processing and statistical analysis), using the FluoroSNNAP software (Patel et al., 2015), we were able to quantify the network’s connectivity in the described conditions (**Table 1**) by inferring functional connectivity of multi-neuron recordings.

The connectivity map and color maps for the BrainPhys medium (**Figure 4E**) and BrainPhys medium with caffeine (**Figure 4H**) show connections in the whole network. In contrast, we only observed activity within the zone optically stimulated for the Neurobasal medium (**Figure 4F**) and BrainPhys medium with synaptic blockers (**Figure 4G**). Additionally, we observed a significantly lower mean global connectivity for the neurons cultured in Neurobasal medium compared to neurons cultured in the BrainPhys medium (*p* ≤ 0.0001) (**Figure 4D**). The mean global connectivity was significantly enhanced with the addition of caffeine compared to the BrainPhys medium condition (*p* ≤ 0.0001) (**Figure 4D**). The addition of synaptic blockers to the BrainPhys medium significantly decreased the mean global connectivity compared to the BrainPhys medium condition (*p* ≤ 0.0001) (**Figure 4D**).

To conclude, we have shown that the quantification of the mean global connectivity using this assay reflects the previously published results in regard to the synaptic connections in Neurobasal (Bardy et al., 2015), synaptic blockers (Lin et al., 2002), and caffeine conditions (van Aerde et al., 2015). Overall, these experiments highlight the sensitivity of our OptoCaMP all optical assay and its potential application in *in vitro* studies aiming to evaluate the neuronal activity in conditions where the neuronal connectivity is enhanced or decreased.

## Discussion

In this paper, we have presented an all optical strategy for quantifying the connectivity of a neural network, using temporal stimuli protocols and OptoCaMP, a combination of a light-activated ion channel, CheRiff and a GECI, jRCaMP1b. The lack of spectral overlap between the optogenetic excitation and jRCaMP1b emission wavelengths enabled the simultaneous stimulation and recording of evoked calcium events in dissociated neuronal cultures *in vitro*. Moreover, this all-optical approach allows to simultaneously record the activity of a large neuronal population with single-neuron readout, therefore allowing the quantification of a neural network’s connectivity. Specifically, it allowed us to simultaneously stimulate a sub-section of a neural network, while recording evoked calcium events in stimulated and non-stimulated neurons. Furthermore, we have demonstrated the capacity of our system to investigate the connectivity of a network under conditions where it is either depressed or enhanced.

The combined stimulation and calcium imaging on a wide-field of view but with single-neuron readout, shown that an increase in the pulse duration (from 10 to 500 ms) resulted in a significant increase in the fluorescence peak. Importantly, the excitation of CheRiff evoked significant higher calcium activity until a plateau was reached using a 500 ms pulses protocol at high intensity stimulation (>25 mW/cm^2^), therefore suggesting saturation of the calcium for long pulses, high blue light intensities. These findings demonstrate the high sensitivity of the channelrhodopsin variant used in OptoCaMP and emphasize a preferable use of low range stimuli intensities.

To select a protocol to study neural networks, we applied protocols using 10, 100, 250, and 500 ms stimulation to a sub-section of the neural network while simultaneously recording the activity of stimulated and non-stimulated neurons. We observed that the 10 ms stimulation evoked similar calcium events in the stimulated and non-stimulated neurons for stimuli intensities from 0 to 44 mW/cm^2^ but not above 44 mW/cm^2^ showing that this protocol enables stimulation and reporting of the neural network activity, but also hinting at potential difficulties with reproducibility at higher light intensities. Although we observed a significant increase in the activity of the stimulated neurons with an increase of the pulse duration, we did not observe any significant difference for the non-stimulated neurons with the 10, 100, and 250 ms protocol. This finding implicates that longer stimuli do not result in higher calcium activity, but it also suggests that the spread of excitation does not depend on the stimuli up to 250 ms pulses duration. In contrast, the 500 ms pulses protocol shown a lower calcium activity in the non-stimulated neurons in zone 2 (red) as compared to zone 1 (green), suggesting that strong stimulus evoke excessive Ca^2+^ activity which has been linked to neuronal damage (Mark et al., 2001). This affects the spread of excitation resulting in the abolition of calcium events in the network, a situation that would impede the study of network connectivity. These observations together with the possibility to increase the throughput, led us to select the100 ms pulses as the optimal pulse duration in this system.

We demonstrate that the use of OptoCaMP along with the stimulation of a sub-section of a neural network enables quantification of a network’s connectivity. Using this technique in various conditions, we observed that the addition of caffeine and synaptic blockers, respectively resulted in a significant increase and decrease of the mean global connectivity as compared to the BrainPhys medium control condition. These results agree with the documented increase of the neuronal excitability with caffeine (Yanovsky et al., 2006; van Aerde et al., 2015) and depression with the addition of synaptic blockers (Lin et al., 2002). Additionally, we observed a significant lower mean global connectivity in the Neurobasal condition as compared to BrainPhys medium which also agrees with previous published findings showing that BrainPhys supports optimal action potentials and synaptic activity, while Neurobasal medium reduces synaptic communication and action potential firing (Bardy et al., 2015). These results demonstrated the sensitivity of our system to report such changes and bring the promise to enable the study of more complex pharmacological conditions. For example, our system could be easily integrated with hardware platforms used for quantitative *in vitro* high-throughput screening and could potentially be adapted to multi-well plates. Under these conditions, it would take ∼10 s to record from each field of view in triplicate, which would correspond to approximatively 30 s/well implying 50 min for a 96 well-plate. This capability could be achieved using automated liquid handlers and other currently available automated platforms to investigate how diseased neural networks communicate and respond to potential therapeutic agent (Agus and Janovjak, 2017). In addition, it is worth mentioning that the use of a smaller magnification high numerical aperture objective (e.g., 10× 0.5 NA), a larger field of view and thus a bigger neuronal network, could also be investigated and analyzed. Moreover, even though the OptoCaMP allows simultaneous stimulation and calcium imaging with single-neuron resolution and readout, the microscope setup used in this study can be considered as a limiting factor as it does not enable the excitation of CheRiff with a micrometer-precise blue illumination pattern (a lever-actuated iris diaphragm was used). To achieve precise stimulation of single-neuron expressing OptoCaMP *in vitro*, the optical system could be adapted with the addition of more precise optical devices, such as a Digital Micromirror Device (Barral and Reyes, 2017). As future possibilities, this system could be extended *in vivo* with the use of single-cell two-photon optogenetic photostimulation (Packer et al., 2015). In this context, ratio-metric calcium imaging would potentially provide a more accurate quantification than a single-wavelength indicator (Thestrup et al., 2014). However, to achieve this goal, the development a Genetically Encoded Ratiometric Calcium Indicator version of jRCaMP1b based on the same approach as the GCaMP-R family would be necessary (Cho et al., 2017). Though notably, previous studies have reported aberrant neuronal activity in GCaMP6-expressing transgenic mouse lines (Steinmetz et al., 2017) indicating that potential OptoCaMP-expressing transgenic mouse lines would require further investigation to evaluate any aberrant activity.

## Author Contributions

WAS conceived and conducted the experiments, analyzed the results, and wrote the manuscript. FG contributed to the cloning strategies and imaging. MD contributed to the Labview programming. FG-M reviewed and helped in the production of the manuscript. MA supervised the project, contributed to the experiments design and data analysis, and reviewed the manuscript.

## Conflict of Interest Statement

The authors declare that the research was conducted in the absence of any commercial or financial relationships that could be construed as a potential conflict of interest.
