# Supplementary material for: All-Optical Assay to Study Biological Neural Networks
Source: Front Neurosci. 2018 Jul 5;12:451. doi: 10.3389/fnins.2018.00451 (PMC6041400; doi:10.3389/fnins.2018.00451)
Supplement: Supplementary file 1 [file Image_1.PDF]

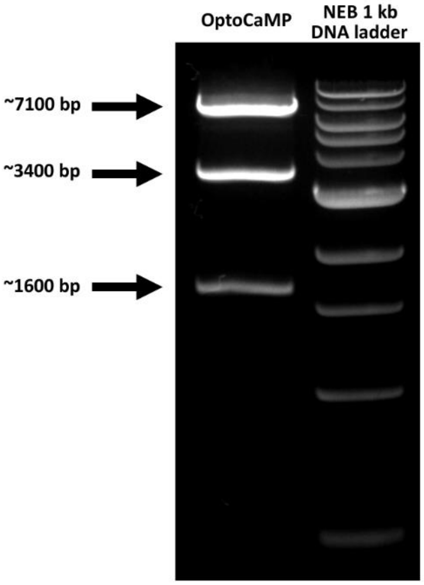

jRCaMP1b-P2A-CheRiff

MLQNELALKLAGLDINKTGGGSHHHHHHGMASMTGGQQMGRDLYDDDDKDLATMVDSS
RRKWNKWGHAVRAIGRLSSANNTEMMYPADGGLRGYTHMALKVDGGGHLSCSFVTTYRS
KKTVGNIKMPAIHYVSHRLERLEESDNEMFVVQREHAVAKFVGLGGGGGTGGSMNSLIKEN
MRMKVVLEGSVNGHQFKCTGEGEGNPYMGTQTMRIKVIEGGPLPFAFDILATSFMYGSRTFI
KYPKGIPDFFKQSFPEGFTWERVTRYEDGGVITVMQDTSLEDGCLVYHVQVRGVNFPSNGA
VMQKKTKGWEPTDSQLTEEQIAEFKEAFSLFDKDGDGTITTKEMGTVMRSLGQNPTEAELQ
DMINEVDADGDGTIDFPEFLIMMAGKMKYTDSEEEIREAFGVFDKDGNGYISAAELRHVMT
NLGEKLTDEEVDEMIREADSDGDGQVNYEEFVQMMTAKRRAGSGATNFSLLKQAGDVEEN
PGPGGAPAPDAHSAPPGNDSAGGSEYHAPAGYQVNPPYHPVHGYEEQCSSIYIYYGALWEQ
ETARGFQWFAVFLSALFLAFYGWHAYKASVGWEEVYVCSVELIKVILEIYFEFTSPAMLFLY
GGNITPWLRYAEWLLTCPVILIHLSNITGLSEAYNKRTMALLVSDLGTICMGVTAALATGWV
KWLFYCIGLVYGTQTFYNAGIIYVESYYIMPAGGCKKLVLAMTAVYYSSWLMFPGLFIFGPE
GMHTLSVAGSTIGHTIADLLSKNIWGLLGHFLRIKIHEHIIMYGDIRRPVSSQFLGRKVDVLAF
VTEEDKVAAAKSRITSEGEYIPLDQIDINVVSKGEELFTGVVPILVELDGDVNGHKFSVSGEG
EGDATYGKLTLKFICTTGKLPVPWPTLVTTLTYGVQCFSRYPDHMKQHDFFKSAMPEGYVQ
ERTIFFKDDGNYKTRAEVKFEGDTLVNRIELKGIDFKEDGNILGHKLEYNYNSHNVYIMADK
QKNGIKVNFKIRHNIEDGSVQLADHYQQNTPIGDGPVLLPDNHYLSTQSALSKDPNEKRDH
MVLLEFVTAAGITLGMDELYK*

Supplementary Figure 1. Agarose gel electrophoresis of the OptoCaMP plasmid. (FCK-CamKII-jRCaMP1b-P2A-CheRiff) digested with the restriction enzymes KpnI and XmaI. Expected and confirmed bands: ~7100 bp, ~3400 bp, ~1600 bp; bp=base pairs. Sequence of OptoCaMP (FCK-CamKII-jRCaMP1b-P2A-CheRiff).
