# Supplementary material for: All-Optical Assay to Study Biological Neural Networks
Source: Front Neurosci. 2018 Jul 5;12:451. doi: 10.3389/fnins.2018.00451 (PMC6041400; doi:10.3389/fnins.2018.00451)
Supplement: Supplementary file 2 [file Image_2.PDF]

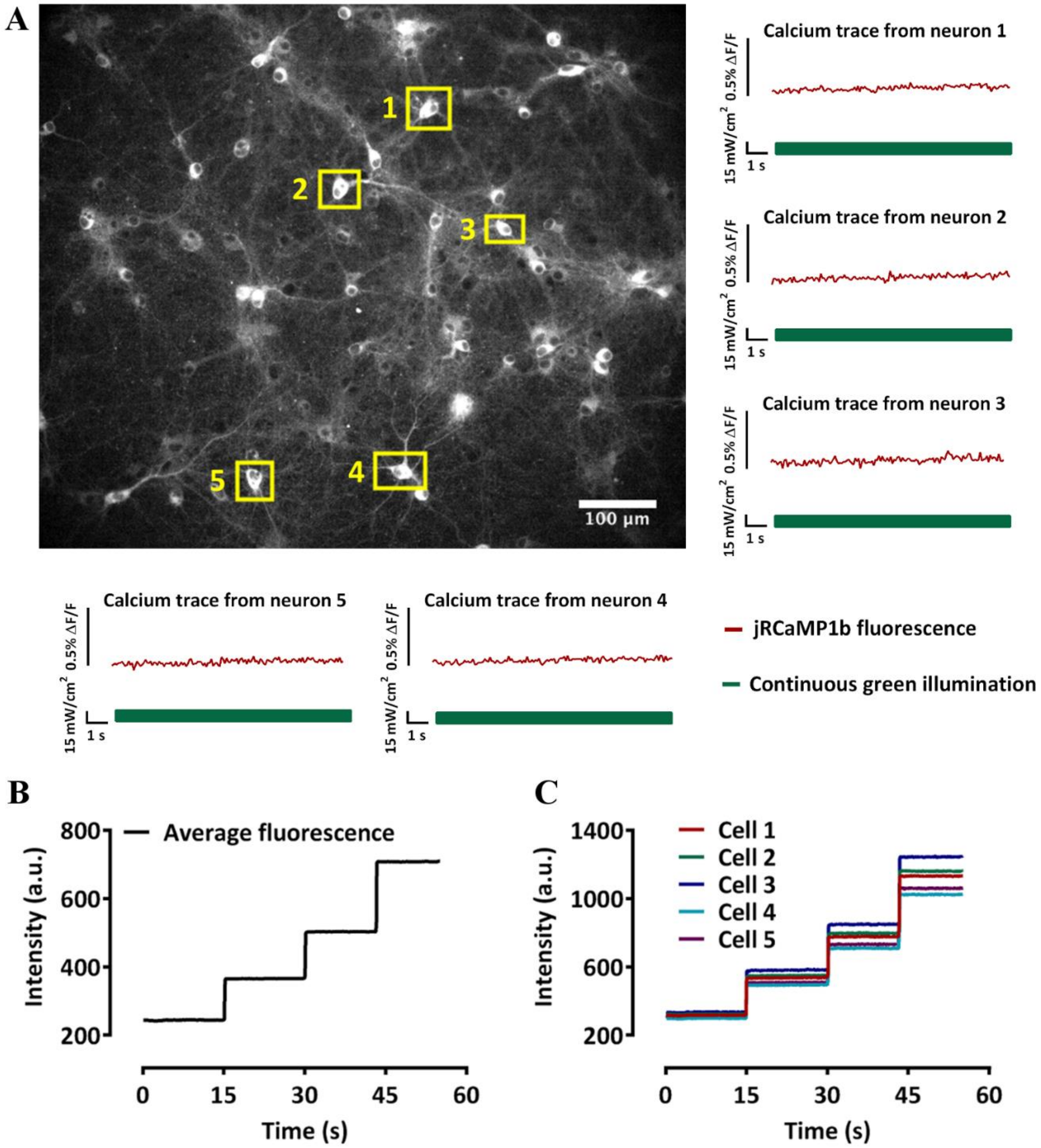

Supplementary Figure 2. Cheriff sensitivity under continuous green illumination at 550 nm. **(A)** Fluorescence image of the microscope field of view (Nikon 20x 0.75 NA air objective) showing a rat cortical neurons culture expressing OptoCaMP (only the mRuby-jRcaMP1b channel is shown) and traces of the change of the GECI jRCaMP1b (red) of individual neurons under continuous green illumination. **(B, C)** To experimentally confirm that the green illumination is not activating Cheriff, we exposed the neurons to steps of continuous green illumination (550 nm) with light intensities below (at 3% and 5%) and above (at 10% and 12%) the power used in the all-optical assays (7%. 15mW/cm^2). Graphs showing the recorded calcium activity (OptoCaMP) of the neurons in the field of view (A): **(B)** Averaged fluorescence signal from the whole field of view and **(C)** raw data from the single cells highlighted in (A). From the graphs, it is visible that green illumination at 550 nm does not induce any detectable calcium activity even with a 12% green light power.
