# Supplementary material for: All-Optical Assay to Study Biological Neural Networks
Source: Front Neurosci. 2018 Jul 5;12:451. doi: 10.3389/fnins.2018.00451 (PMC6041400; doi:10.3389/fnins.2018.00451)
Supplement: Supplementary file 3 [file Image_3.PDF]

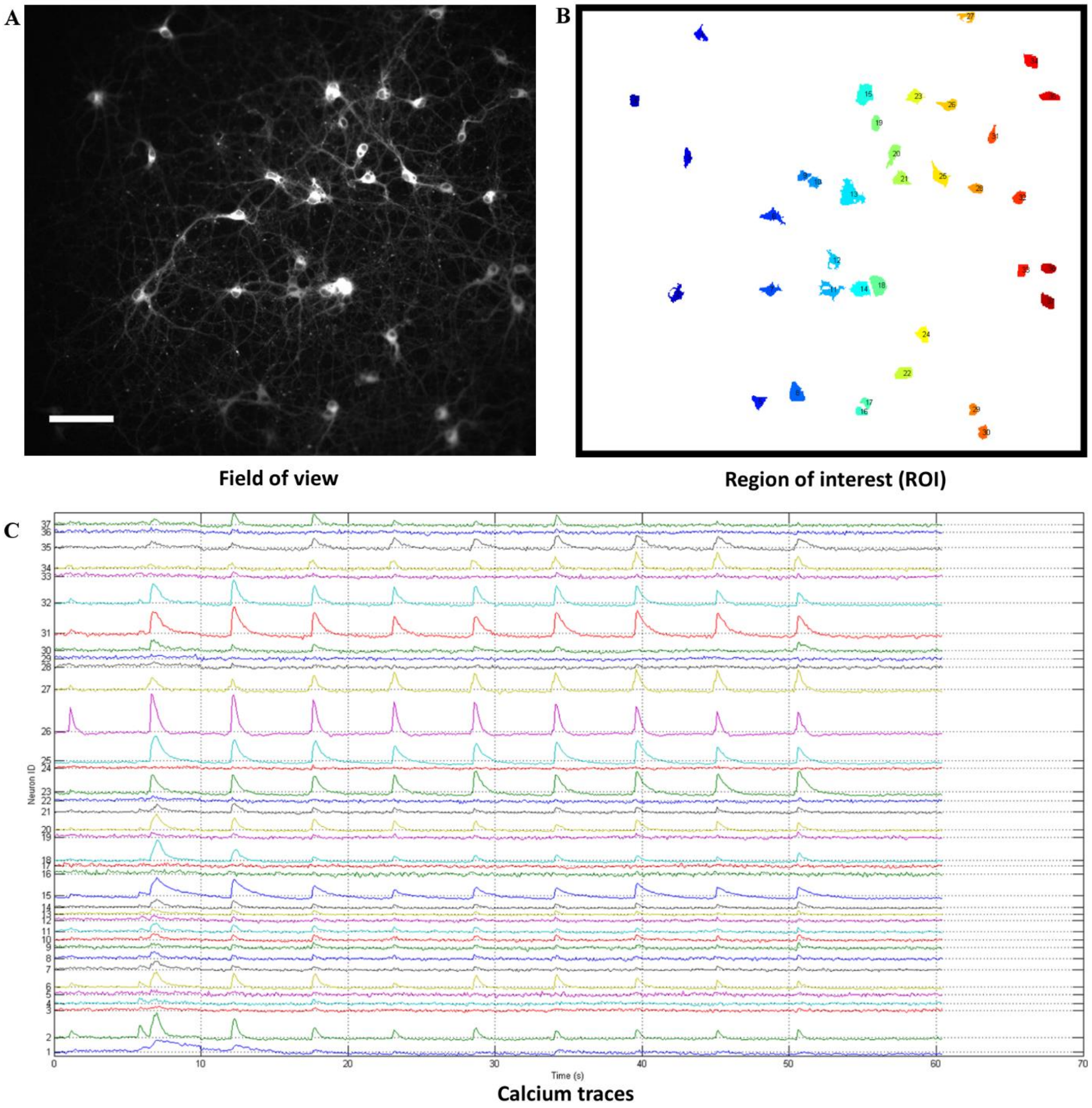

Supplementary Figure 3. Workflow using FluoroSNNAP.
(A) Fluorescence image (mRuby channel - jRCaMP1b) of the field of view analysed. **(B)**. Following the segmentation step, the calcium trace from individual neurons (region of interest) is extracted **(C)** and used for the further analysis (e.g. mean global connectivity).
